# Combined Management of Apical Root Fracture and Avulsion of Two Maxillary Permanent Central Incisors: A Case Report

**DOI:** 10.3390/dj9040039

**Published:** 2021-04-01

**Authors:** Giulia Bardini, Davide Musu, Silvia Mezzena, Claudia Dettori, Elisabetta Cotti

**Affiliations:** Department of Conservative Dentistry and Endodontics, University of Cagliari, 09125 Cagliari, Italy; davidemusu.dds@gmail.com (D.M.); silvia@studio-mezzena.it (S.M.); claudia.dettori@gmail.com (C.D.); cottiendo@gmail.com (E.C.)

**Keywords:** dental trauma, extrusive luxation, replantation, root fracture, crown fracture

## Abstract

As a result of a skiing accident, a ten-year-old girl suffered combined injuries to both maxillary central incisor teeth (#1.1 and #2.1). The injuries were uncomplicated crown fractures, apical horizontal root fractures, and a severe extrusive luxation of the coronal segments of the teeth. Her mother repositioned the teeth immediately, resulting in good initial healing. Nine months later, the patient was referred to a specialist to manage the endodontic consequences of the trauma. The apexification treatment of the fractured roots, using a preformed apical barrier technique with bioactive cement, was the treatment of choice, administered to both the avulsed roots at two separate recall visits. The best option for managing the fractured apical segments was to continue with the follow-up, which was conducted to assess the overall case at 30 months. The fractured apexes remained normally positioned inside the socket and were asymptomatic (as they presumably maintained a physiological vascular-nerve supply and, consequently, their vitality), while the apexification treatment led to the healing of the periodontal tissues and to hard tissue formation in the area of the interrupted roots in the avulsed portion of the teeth. The management of traumatic injuries in teeth often requires multiple treatment approaches, because these injuries rarely represent one single type of trauma.

## 1. Introduction

Traumatic injuries to the stomatognatic system occur frequently, and represent 6–34% of all traumatic events within the population [1,2]. Reportedly, 50% of dental trauma affects dental hard tissue, while 36% also involves the soft tissues of the oral cavity [3].

Dental trauma is common during childhood and adolescence, and the most frequent causes for its insurgence are accidental falls and collisions during sport activities [1,2]. Maxillary central incisors are the teeth most often involved, followed by upper laterals. Most importantly, teeth frequently sustain a combination of several injuries [1,2].

The avulsion of permanent teeth represents 0.5–3% of all dental traumas [4,5], and especially involves anterior teeth in children aged 9–11 years. The prognosis of this injury, which entails damage to the vascular-nerve bundle and to the surrounding periodontal tissues, depends on the adequacy of the emergency treatment performed and on the timeliness with which it is completed [6,7,8,9,10,11]. The gold standard to treat this injury is the immediate replantation of the avulsed tooth (within one hour from the accident), which may successfully facilitate the reattachment of the periodontal ligament, and offer a good long-term outcome [1,2]. When the repositioning takes place more than 60 min after the avulsion, it is called “delayed replantation”, and its prognosis is limited, because of the increased risk of resorptive and ankylosis-related complications in the root [1,2,11]. Furthermore, root fractures are also complex injuries involving all dental tissues, and are relatively rare compared to other dental injuries, their frequency being about 0.5–7% in permanent teeth [12]. To facilitate the healing of root fractures, it is necessary to optimally reposition the coronal fragment of the root in its socket [13]. The treatments for both fractures and extrusive luxations require flexible splinting to the adjacent teeth for an average of 4 weeks, and meticulous follow-up to assess the conditions of the pulp in terms of vitality and the roots in terms of resorption [2,13,14]. It has been reported that a successful treatment can be obtained in approximately 80% of cases [12].

If any pathological sign or symptom develops, the appropriate endodontic treatment should be performed on the apical end of the coronal segment [15]. 

This article aims to report on the combined management of two permanent maxillary central incisors that underwent severe extrusive luxation, following the double fracture of the apical third of their roots.

## 2. Case Report

**Figure 1:**
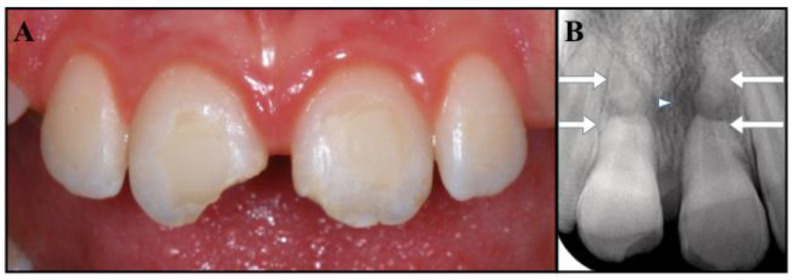
(**A**) Clinical photograph showing the uncomplicated crown fracture of the incisal third of teeth #1.1 and #2.1. (**B**) Periapical radiograph showing the apical root fracture of teeth #1.1 and #2.1 (arrowed) and the radiolucency at the fracture line of #2.1 (small arrow).

A 10-year-old Caucasian girl suffered combined injuries to both maxillary central incisors while skiing. At the moment of the traumatic event, her mother was able to collect the extruded portions of the two teeth and to perform the immediate repositioning of both segments. The patient was then taken to a general dentist to assess the status of the teeth involved in the accident. The patient’s medical history was noncontributory. Following the clinical examination, the dentist reported that teeth #1.1 and #2.1 had suffered extrusive luxation, had been replanted correctly, and that tooth #1.1 also had an enamel–dentin uncomplicated crown fracture, while tooth #2.1 exhibited a fracture limited to the enamel. Damages to the alveolar bone, or to the soft tissues, were not observed. The dentist applied a passive and flexible splint for 4 weeks; wire and composite stabilization were chosen so as to favor oral hygiene and because they are well tolerated. Furthermore, the dentist prescribed antibiotics (Amoxicillin with clavulanic acid, 1000 mg, twice a day, for one week) and advised the patient to take care of the injured teeth and to rinse with an antibacterial agent (chlorhexidine gluconate 0.12%). The dentist monitored the teeth, which remained asymptomatic, and nine months after the trauma the dentist referred the patient to an endodontist to check the pulpal status of the traumatized elements. During the consultation, the girl reported a mild tenderness to pressure on tooth #2.1. The soft tissues appeared healthy; the extruded teeth showed no signs of discoloration, grade 1 mobility, and a coronal fracture on both teeth. Tooth #2.1 was tender to percussion and palpation and did not respond to pulp sensitivity tests, while tooth #1.1 was slightly tender to percussion and did respond to sensitivity tests. Interestingly, the periapical radiographs showed the presence of a horizontal fracture of the apical third in both the avulsed teeth. The periapical area of the coronal fragment of tooth #2.1 showed a defined radiolucency, while no clear radiolucency was visible on the fractured portion of tooth #1.1 (Figure 1B).

The diagnosis was as follows: apical horizontal root fractures of teeth #1.1 and #2.1; severe extrusive luxation of the coronal fragment of both central incisors, which were successfully repositioned; pulp necrosis and chronic apical periodontitis on the coronal portion of tooth #2.1; uncomplicated crown fractures of the incisal third of teeth #1.1 and #2.1. The residual apexes did not show signs or symptoms of apical periodontitis (Figure 1A,B). The prognosis was considered guarded. The proposed treatment was apexification on tooth #2.1 (as the fractured portion of the coronal fragment mimicked an open apex), restoration of the access cavity and of the crown fractures with bonded composite resin, and follow-up of the overall case. The patient and her parents were informed of the diagnosis, treatment plan alternatives, and prognosis of the teeth. Written consent was obtained, and the proposed endodontic management was accepted.

**Figure 2:**
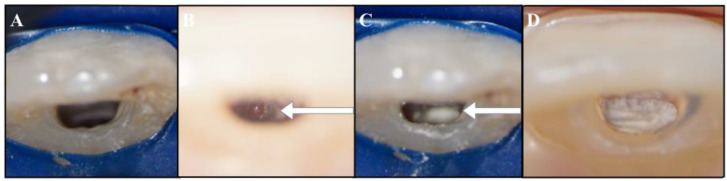
(**A**) Standard access cavity of tooth #2.1. (**B**) Identification of the wide opening of the apical portion of the coronal fragment of tooth #2.1 (arrowed). (**C**) Apical barrier of *Portland* cement (arrowed). (**D**) Complete obturation of the canal with the cement.

After the administration of local anesthesia, a rubber dam was put in place, and a standard access cavity was opened to perform the endodontic treatment on tooth #2.1 (Figure 2A). The working length was assessed with an intra-operative periapical radiograph with a #25 hand k-file, and the canal was instrumented using a sequence of hand K-files of alternating irrigation and with a copious amount of 5.25% sodium hypochlorite. The apical portion of the coronal segment of the root was examined under a Zeiss surgical microscope (Oberkochen, Germany), and the wide opening was identified as shown in Figure 2B.

Upon finishing the instrumentation, calcium hydroxide was introduced as an intracanal medication for 4 weeks, and the access cavity was closed with a cotton pellet and temporary cement (Cavit, 3M ESPE, St. Paul, MN, USA). The tooth was asymptomatic during the postoperative period.

**Figure 3:**
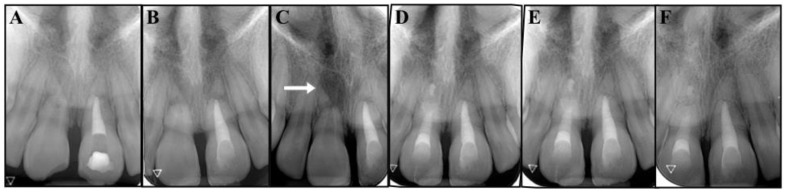
(**A**) Root canal filling with of tooth #2.1. (**B**) Post obturation and restoration of tooth #2.1. (**C**) The 6-month follow-up periapical radiograph reveals the radiolucency of tooth #1.1 (arrow). (**D**) Root canal filling with Portland cement and resin restoration of tooth #1.1. (**E**) Image from the 12-month follow-up. (**F**) Image from the 30-month follow-up.

At the second appointment, the temporary filling was assessed as intact, the procedure was repeated, the calcium hydroxide was removed, and the canal was filled with Portland cement (ProRoot MTA, Dentsply Tulsa Dental, Tulsa, OK, USA) (Figure 2C,D) [16,17,18]. The cement was prepared according to the manufacturer’s instructions, carried into the canal with the MAP System (MicroApical Placement Dentsply, Maillefer, Switzerland) and condensed with hand pluggers. The access cavity was sealed with a composite bonded restoration [19]. Two class IV restorations were further performed on the fractured coronal segments of teeth #1.1 and #2.1, using composite and dental adhesive (Figure 3B).

Six months later, tooth #1.1 no longer responded to the sensitivity tests and the periapical radiograph showed a radiolucent area at the fracture site of the root (Figure 3C).

Tooth #1.1 was diagnosed with pulp necrosis and apical periodontitis in the coronal fragment, and an apexification procedure was performed with the same protocol as the one previously described for tooth #2.1 (Figure 3D). We then filled the canal with Portland cement (Aureoseal, Ogna lab, Muggiò, Italy).

**Figure 4:**
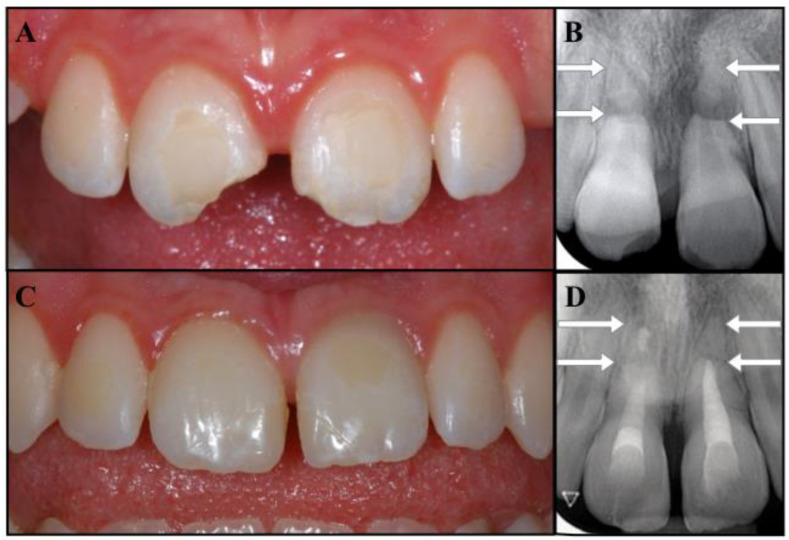
(**A**,**B**) Clinical photograph and intraoral radiographs of teeth #1.1 and #2.1 at the initial stage of treatment (replantation) and at the 30-month follow-up. (**C**,**D**) The images show good coronal restorations, mild signs of discoloration in the cervical portion of tooth #2.1, healing in the fracture lines, a healthy periapex.

The follow-up was carried out according to schedule. At 30 months, the patient was free of symptoms, and the periapical radiographs showed healthy periodontal tissues around both the fractured roots and the apical fragments, while the fractured areas seemed to have healed, with hard tissue deposition (Figure 4C,D). The tooth eruption was assessed to be normal with the continued development of the alveolar ridge. Undesirably, tooth # 2.1 showed mild signs of discoloration. The patient was informed that the clinical and radiographic follow-up would continue once a year for at least another three years, and ideally for as long as possible. She was also made aware that further considerations will be necessary upon completion of the permanent dentition.

## 3. Discussion

The complete extrusive luxation of permanent teeth is a real dental emergency, and dentists should always be adequately prepared to manage this situation [1,2].

According to the recently updated guidelines from the International Association of Dental Traumatology (IADT) [2], the immediate repositioning of the coronal fragment is the treatment of choice and should be performed at the site of the accident if possible [1,2]. When immediate repositioning is not feasible, the alternative is to provide a delayed replantation. Physiologic fluids to be used for the storage and transportation of the avulsed tooth include tissue culture and cell transport media. Examples of osmolarity-balanced media include HBSS, saline and milk, and saliva can also be used [20,21]. Delayed replantation compromises long-term prognosis, due to the high probability of the periodontal ligament becoming necrotic. In this case, the expected mid- to long-term outcome is ankylosis and root resorption, with eventual loss of the tooth [22]. Nevertheless, replantation should always be attempted, because in the short- to medium-term, it will help preserve the alveolar bone, the esthetics, and the function of the dental element, while improving the psychological status of the young patient. Following replantation, passive and flexible splinting should be applied for 2 weeks so as to immobilize and maintain the tooth in the correct position in the initial period, and to provide comfort and improved function [2]. In this case, the dentist applied a passive and flexible splint for 4 weeks, because of the combined injuries on both maxillary central incisors.

Radiographs are of paramount importance in managing dental injuries, and conventional two-dimensional images are usually a good standard for appropriate diagnosis and treatment planning, while offering a baseline for comparison at follow-ups [13]. Cone beam computerized tomography (CBCT) can be considered for use in specific injuries [13], as it provides greater detail in terms of the location, extension and direction of root fractures. The concern is that children aged 11–15, exposed to CBCT examinations, exhibit significantly higher levels of individual cumulative dose than other age groups, and there is thus an increased life attributable risk (LAR) for children under 15 years of age [23]. It is therefore advisable to not expose a young patient to additional ionizing radiations unless considered necessary [23]. In this case, the gap between the root fragments made it possible to clearly detect the root fractures on conventional radiographs [24], and the additional information obtained from the 3D image did not necessitate a change in the management of the injury.

The next step is assessing the endodontic status of traumatized teeth and planning a coherent therapy. While in cases of delayed replantation conventional root canal treatment is always necessary, the choice of endodontic therapy after immediate replantation depends on the degree of maturity of the root (open or closed apex) and the condition of the periodontal ligament cells [1,2]. In the avulsed permanent tooth with an open apex, the goal is to wait for the possible spontaneous revascularization of the pulp space [25], in order to allow the further development and strengthening of the root [26]. In case revascularization does not occur, a following apexification treatment may be necessary [27]. The recommended treatment for a fractured root exhibiting extrusive luxation of its coronal portion also requires the repositioning of the coronal fragment, as well as short-term, passive and flexible splinting. Pulp necrosis is a possible late complication that usually occurs in the coronal segment of the fracture, even though spontaneous revascularization may occur in immature teeth. In general, the apical fragment rarely undergoes pulp necrosis [13,28]. Apexification with a preformed apical barrier (MTA) has become a reliably successful procedure [29] for open apexes both in real immature teeth and in fractured roots [30,31,32]. The role of hydraulic and bioactive cement in this process is established by its potential to induce hard tissue formation, which leads to an effective immediate seal and to a further apical barrier [16,17,33].

However, the thin dentinal walls of the roots still present a clinical problem, particularly in cases involving a subsequent traumatic event, as these teeth would easily fracture [28]. On the other hand, for avulsed, mature, permanent teeth, the ideal time to begin root canal treatment is 7–10 days after replantation, and before splint removal [1,2].

When two types of injury occur simultaneously on the same tooth, the resulting negative synergistic effect will lead to a more unfavorable outcome [13].

The complexity of this case derives from the combination of root fractures with the avulsion of the coronal fragments, which places the traumatic injury into two different categories (Figure 1B). Fortunately, it was possible to merge the recommendations [1,2,13] and provide a reasonable treatment plan. At 30 months, the patient was asymptomatic, the replanted fragments did not exhibit mobility or a metallic percussion sound, and radiographs showed no evidence of infection-related or ankylosis-related resorption. The application of an apexification treatment to the coronal segments has enabled the healing of apical periodontitis and the repair of the fractured ends of the roots, while the apexes seem to have maintained their vitality, as shown by the lack of symptoms and clinical and radiographic signs (Figure 4C,D). The only unfavorable outcome was represented by a mild discoloration detected in the cervical area of tooth # 2.1.

This undesirable outcome may be caused by the use of calcium silicate cements in the apexification procedure [34,35], when they contain bismuth oxide as a radiopacifier [34,35,36]. The proper choice and the way to use a *Portland* cement may reduce the chances of this aesthetic consequence [34].

Hypothetically, if the apical fragments become non-vital in the future, they could be surgically removed [37,38,39,40].

## 4. Conclusions

When a traumatic event occurs, teeth often sustain a combination of injuries. We reported on the present case in order to discuss the attitude that a clinician may have in merging the protocols available from the different aspects of traumatology, endodontics, periodontics, and restorative dentistry, involving good timing and the right indication. This attitude may simplify the decision-making process when supported by competent knowledge, and thus enable the implementation of a coherent and successful treatment plan.

## Data Availability

Not applicable.
